# Healthcare‑associated infections in intensive care unit patients with and without COVID-19: a single center prospective surveillance study

**DOI:** 10.1186/s13756-023-01353-6

**Published:** 2023-12-18

**Authors:** Nando Bloch, Susanne Rüfenacht, Magdalena Ludwinek, Waldemar Frick, Gian-Reto Kleger, Florian Schneider, Werner C. Albrich, Domenica Flury, Stefan P Kuster, Matthias Schlegel, Philipp Kohler

**Affiliations:** 1 Division of Infectious Diseases and Hospital Epidemiology, Cantonal Hospital St.Gallen, St.Gallen, Switzerland; 2 Division of Intensive Care, Cantonal Hospital St.Gallen, St.Gallen, Switzerland

**Keywords:** Healthcare-associated Infection, Intensive care unit, COVID-19, Surveillance, COVID-19 burden

## Abstract

**Background:**

The coronavirus disease 2019 (COVID-19) pandemic led to a global increase in healthcare-associated infections (HAI) among intensive care unit (ICU) patients. Whether this increase is directly attributable to COVID-19 or whether the pandemic indirectly (via staff shortages or breaches in infection prevention measures) led to this increase, remains unclear. The objectives of this study were to assess HAI incidence and to identify independent risk factors for HAI in COVID-19 and non-COVID-19 ICU patients.

**Methods:**

We established a monocentric prospective HAI surveillance in the medical ICU of our tertiary care center from September 1st 2021 until August 31st 2022, during circulation of the SARS-CoV-2 delta and omicron variants. We consecutively included patients ≥ 18 years of age with an ICU length of stay of > 2 calendar days. HAI were defined according to the European Centre for Disease Prevention and Control definitions. HAI rate was calculated per 1,000 patient-days or device-days; risk ratios (RR) and corresponding 95% confidence intervals (CI) for COVID-19 versus non-COVID-19 patients were calculated. We used multivariable Cox regression to identify independent risk factors for HAI. As a proxy for institutional COVID-19 burden, weekly COVID-19 density (i.e. percentage of COVID-19 patients among all ICU patients) was included in the model as time-dependent co-variable.

**Results:**

We included 254 patients, 64 (25.1%) COVID-19 and 190 (74.9%) non-COVID-19 patients; 83 HAI in 72 patients were recorded, thereof 45 ventilator-associated lower respiratory tract infections (VA-LRTI) (54.2%) and 18 blood stream infections (BSI) (21.6%). HAI incidence rate was 49.1/1,000 patient-days in COVID-19 and 22.5/1,000 patient-days in non-COVID-19 patients (RR 2.2, 95%-CI 1.4–3.4). This result was mainly due to different VA-LRTI rates (40.3 vs. 11.7/1,000 ventilator days, *p* < 0.001), whereas BSI rates were not statistically different (9.4 vs. 5.6/1,000 patient days, *p* = 0.27). Multivariable analysis identified COVID-19 as main risk factor for HAI development, whereas age, mechanical ventilation and COVID-19 density were not significant.

**Conclusions:**

These data from the fourth and fifth wave of the pandemic show a higher HAI incidence in COVID-19 than in non-COVID-19 ICU patients, mainly due to an increase in pulmonary infections. A diagnosis of COVID-19 was independently associated with HAI development, whereas institutional COVID-19 burden was not.

**Supplementary Information:**

The online version contains supplementary material available at 10.1186/s13756-023-01353-6.

## Background

Healthcare-associated infections (HAI) are a major patient safety and public health concern contributing to an estimated cumulative burden of approximately 500 disability-adjusted life years per 100,000 general population each year in the European Union and the European Economic Area [1]. Intensive care unit (ICU) patients are at highest risk for HAI mainly due to illness severity, exposure to invasive devices and procedures and prolonged length of stay (LOS) [2]. Early in the coronavirus disease 2019 (COVID-19) pandemic, a global increase in HAI incidence was observed. HAI incidence was seen to be coincident with severe acute respiratory coronavirus virus 2 (SARS-CoV-2) surges, thereby improving during low SARS-CoV-2 activity and increasing during high activity [3–5]. This trend lasted also for the second year of the pandemic, as exemplarily reported from the US where a dramatic increase in HAI incidence was observed as the SARS-CoV-2 delta variant emerged in the third quarter of 2021 [6].

Data on HAI incidence and risk factors for developing HAI in COVID-19 patients are highly heterogeneous, rendering it difficult to contextualize them. Heterogeneity could be explained, among others, by the emergence of virus variants, changes in diagnostic practices and treatment, differences in HAI case definitions and lack of concurrent control groups [7, 8]. HAI are described with a higher-than-average incidence compared to that in non-COVID-19 patients before the pandemic, ranging up to 20% in non-critically ill and up to 50% in critically ill COVID-19 patients [9–12]. In a large systematic review, HAI in COVID-19 patients was associated with ICU stay and mechanical ventilation (MV). [7].

In addition to patient-level risk factors, hospital-level risk factors may have contributed to the global HAI increase, made up of the additional burden of care associated with the pandemic, higher acuity patient population or possible disruptions of staffing and infection prevention and control (IPC) measures [3, 13]. How far hospital-level risk factors may have influenced HAI in non-COVID-19 patients and to what extent the global increase in HAI was caused by HAI in non-COVID-19 patients is not well described.

We hypothesized that understaffing and breaches in IPC measures during the pandemic might have at least partially contributed to an increase in HAI incidence among our ICU patients. The objective of this study was therefore to evaluate the local HAI incidence separately for COVID-19 and non-COVID-19 patients contemporaneously hospitalized on our medical ICU and to assess the association of institutional COVID-19 burden with the risk for HAI.

## Methods

### Setting and population

We conducted a prospective HAI surveillance in a 12-bed medical ICU of our tertiary care center from September 1st 2021 until August 31st 2022. SARS-CoV-2 delta was the predominant virus variant from September 2021 until 21th December 2021, when it was gradually replaced by the omicron BA.1 variant [14]. We consecutively included all adult patients (i.e. ≥18 years) with an ICU-LOS of > 2 calendar days. For patients with multiple ICU admissions during a single hospitalization episode, only the first ICU episode was considered.

### Study procedures and definitions

Electronic medical records were screened three times per week by an IPC expert; records of patients with HAI suspicion were additionally reviewed by an infectious disease physician. COVID-19 status at ICU admission (i.e. positive or negative) was our main predictor variable. Further variables included age, sex, disease severity as assessed by the Simplified Acute Physiology Score II (SAPS II) during the first 24 h after ICU admission [15], the date of ICU admission and discharge, antibiotic treatment and ICU mortality. We also recorded the dates of insertion and removal of any central and peripheral lines, urinary catheters, endotracheal tubes and tracheostomies, as well as of start and ending of any MV. Device utilization ratios (DUR) were calculated by dividing the number of device days (central lines, peripheral lines, urinary catheters and MV) by the ICU-LOS.

HAI were defined according to the European Centre for Disease Prevention and Control (ECDC) case definitions [16, 17]. Therein, ICU-acquired infections are defined as occurring > 48 h after admission. After ICU discharge, patients were followed up concerning HAI for 48 h; in case of urinary catheters for 7 days. Device-associated infection is defined as HAI in a patient with a device within the 48-hour period (even intermittently) before onset of infection. Pneumonia (PN) is defined by a combination of clinical symptoms, radiological and laboratory criteria. For our analysis, we summarized PN as well as lower respiratory tract infections others than pneumonia (ECDC case definitions LRI-BRON, LRI-LUNG) as lower respiratory tract infection (LRTI); LRTI were considered ventilator associated (VA-LRTI) when the patient was invasively ventilated within the 48-hour period (even intermittently) before onset of infection. For further information on classification of HAI see Additional File 1. As a proxy for institutional COVID-19 burden, we calculated weekly “COVID-19 density” on the ICU, defined as percentage of COVID-19 patients among all ICU patients.

### Statistical analysis

We used descriptive statistics to compare patient characteristics and DURs according to COVID-19 status, as well as outcome data (i.e. antibiotic usage and ICU mortality). Student’s t-test or Mann-Whitney-U test were used for continuous variables, and chi-square or Fisher’s exact test, as appropriate, for categorical variables. We assessed HAI incidence rate per 1,000 patient-days (for BSI) or per 1,000 device days (for device-related infections); rate ratios (RR) and corresponding 95% confidence intervals (CI) comparing COVID-19 and non-COVID-19 patients were calculated.

For visualization we plotted the cumulative cause-specific hazard of developing a HAI according to COVID-19 status (Nelson-Aalen estimate); log-rank test was used to compare groups. Discharged and deceased patients were censored at time of the event. We used Cox regression to identify independent risk factors for HAI; adjusted hazard ratios (aHR) and corresponding 95% CI were calculated. Besides patient characteristics (sex, age, disease severity), we included MV (excluding MV started after HAI diagnosis), and COVID-19 density (as time-dependent variable) in the model. Variables reaching a *p*-value of ≤ 0.10 in univariable analysis were included in the multivariable model. For the final model, a *p*-value < 0.05 was considered statistically significant. All statistical analysis was performed using R statistical software, version 4.2.1 [18] and OpenEpi Version 3.01 [19]. This manuscript follows the STROBE reporting guideline [20].

## Results

### Patient characteristics

We included 254 patients, 64 (25.1%) COVID-19 and 190 (74.9%) non-COVID-19 patients. COVID-19 patients were younger compared to non-COVID-19 patients (median age 59.9 vs. 65.5 years; *p* < 0.001); median ICU-LOS was 11 days for COVID-19 vs. 5 days for non-COVID-19 patients (*p* < 0.001). DURs for MV and central lines were also higher in COVID-19 compared to non-COVID-19 patients (0.89 vs. 0.56, *p* < 0.001; resp. 0.90 vs. 0.75, *p* = 0.044). Median duration of antibiotic treatment was 9 days for COVID-19 vs. 5 days for non-COVID-19 patients (*p* = 0.001) (Table 1).

**Table 1:** Patient characteristics by COVID-19 status

Characteristics	All patients	COVID-19	non-COVID-19	p
N (%)	254 (100)	64 (25.1)	190 (74.9)	
Male sex – n (%)	172 (67.7)	45 (70.3)	127 (66.8)	0.72
Age - years – median (IQR)	63.3 (53.5–71.8)	59.9 (49.3–65.1)	65.5 (57.4–73.2)	< 0.001
SAPS II – median (IQR)	45.0 (35.0-58.8)	43.5 (34.3–56.5)	46.0 (35.0-59.8)	0.30
Mechanical ventilation DUR – median (IQR)	0.72 (0–1)	0.89 (0.63-1)	0.56 (0-0.96)	< 0.001
Central venous catheter DUR – median (IQR)	0.8 (0–1)	0.90 (0.68-1)	0.75 (0–1)	0.044
Urinary catheter DUR – median (IQR)	1 (0.95-1)	1 (0.97-1)	1 (0.88-1)	0.52
Antibiotic treatment - n (%)	187 (75.7)	52 (82.5)	135 (73.3)	0.14
Days of antibiotic treatment - median (IQR)	5 (1–12)	9 (4–20)	5 (0–10)	0.001
ICU length of stay in days - median (IQR)	6 (5–11)	11 (6-22.3)	5 (4–8)	< 0.001

IQR, interquartile range; SAPS II, Simplified Acute Physiology Score II; DUR, Device Utilization Ratio (device days/patient days); ICU, Intensive Care Unit

### Healthcare-associated Infections

**Fig. 1:**
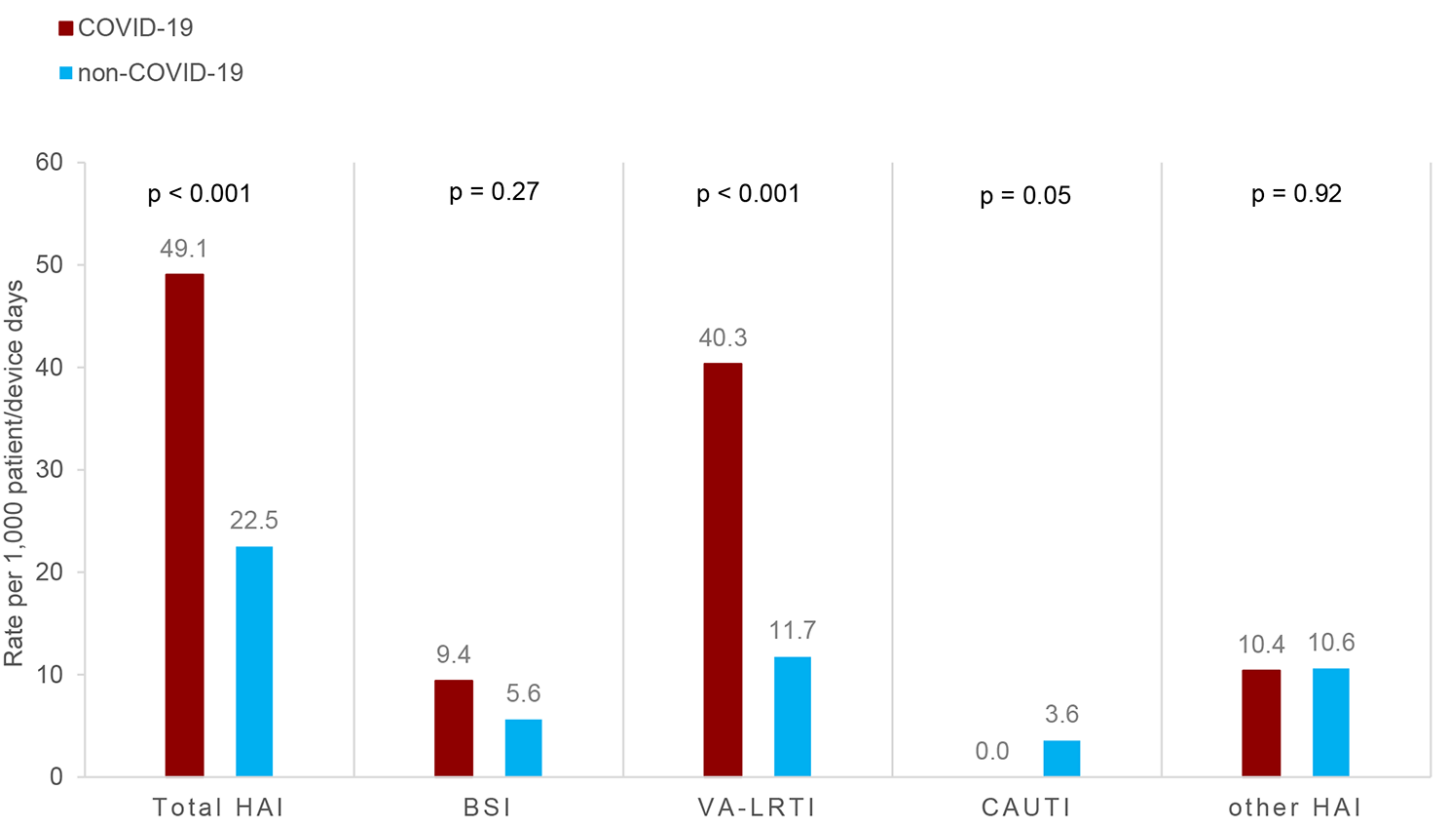
HAI rate per 1,000 device/patient days in COVID-19 vs. non-COVID-19 patients. HAI, health-care associated infections; BSI, blood stream infections (includes central line associated blood stream infections (CLABSI), peripheral vascular catheter infections, and secondary BSI); VA-LRTI ventilator-associated lower respiratory tract infections; CAUTI, catheter associated urinary tract infections; other HAI include non-ventilator-associated lower respiratory tract infections among others

Of the 64 COVID-19 patients, 36 (56.3%) had a total of 47 HAI. In non-COVID-19 patients, 36 of 190 (18.9%) experienced 36 HAI, corresponding to a HAI incidence rate of 49.1/1,000 patient-days in COVID-19 and 22.5/1,000 patient-days in non-COVID-19 patients (RR 2.2, 95% CI 1.4–3.4). This result was mainly due to different VA-LRTI rates (40.3 vs. 11.7/1,000 ventilator days, *p* < 0.001), whereas BSI rates were not statistically significant (9.4 vs. 5.6/1,000 patient days, *p* = 0.27) (Fig. 1).

**Fig. 2:**
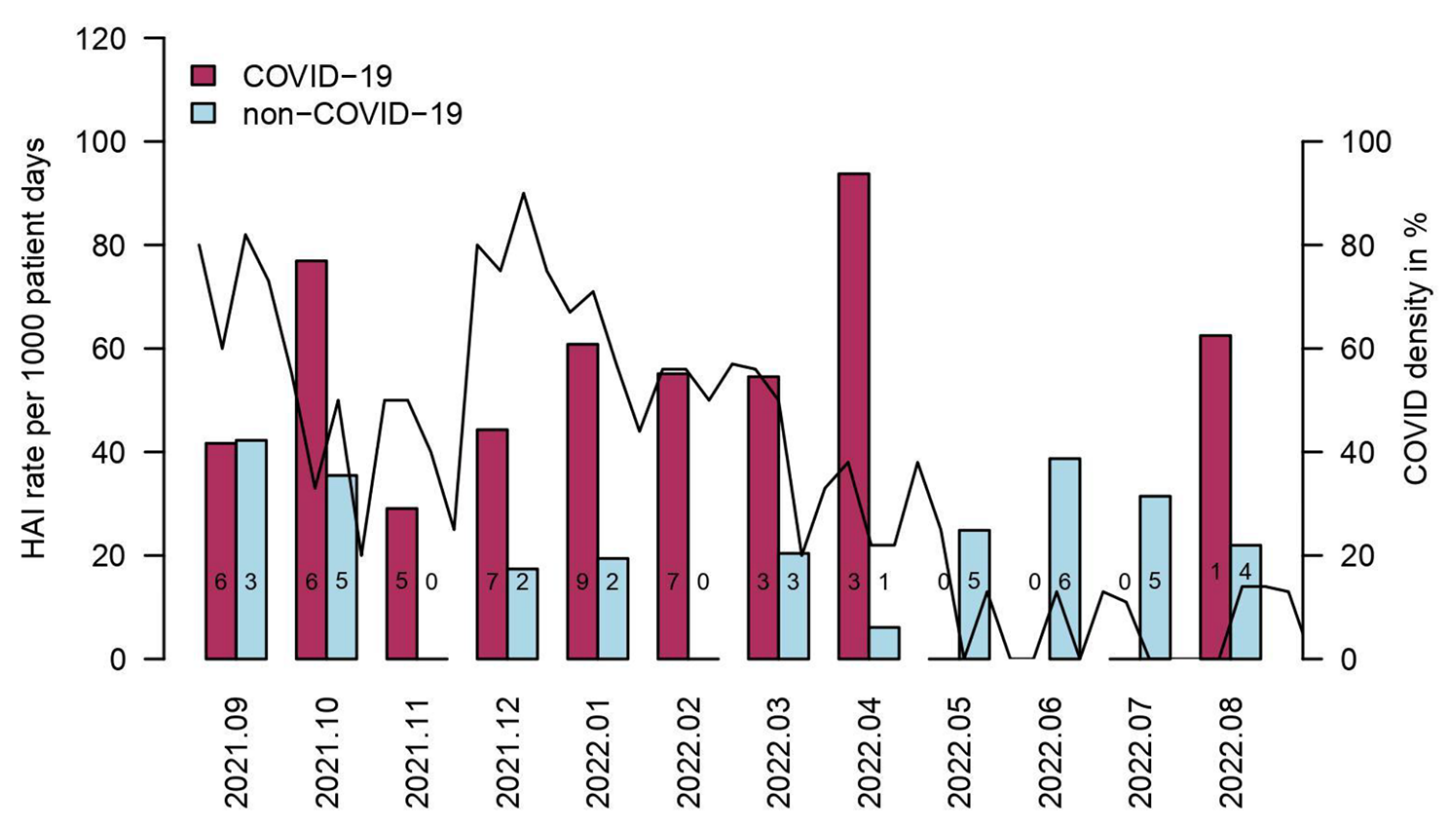
Monthly HAI rates in comparison to COVID-19 density on intensive care unit. HAI, healthcare-associated Infections. X-axis indicates surveillance period from 1st of September 2021 until 31st of August 2022. Y-axis shows monthly HAI rates per 1,000 patient days and black line shows weekly COVID-19 density (percentage of COVID-19 patients on ICU). The numbers in the columns represent the actual number of patients with a HAI in COVID-19 and non-COVID-19 patients

Figure 2 shows monthly HAI rate in COVID-19 and non-COVID-19 patients, as well as weekly COVID-19 density. COVID-19 density ranged from 0% up to 90%; no obvious correlation was observed.

### Survival analysis and Cox regression model

**Fig. 3:**
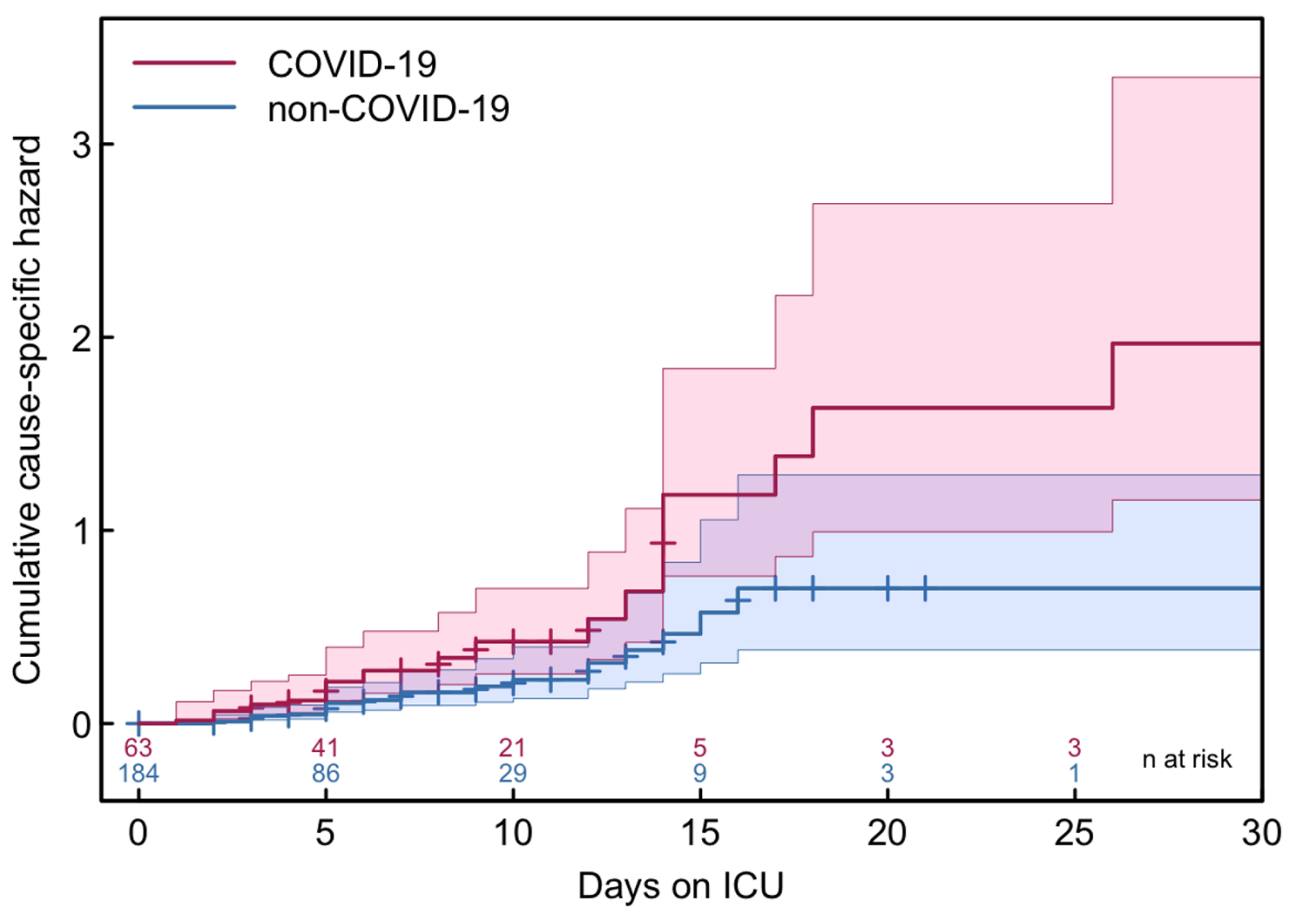
Nelson-Aalen estimate showing the cumulative hazard of developing a HAI in COVID-19 vs. non-COVID-19 patients. Red and blue bands correspond to 95% confidence intervals. Colored numbers on the x-axis represent the actual number of patients a risk at every time point for COVID-19 and non-COVID-19 patients, ICU, Intensive Care Unit

In univariable analysis, COVID-19 positivity was associated with higher risk for HAI (log-rank test *p* = 0.002) (Fig. 3; Table 2).

In the multivariable Cox regression model, COVID-19 status remained the only significant factor associated with increased hazard for HAI (aHR 1.9, 95% CI 1.0-3.6, *p* = 0.045); neither COVID-19 density nor sex, age, or SAPS II were associated with HAI (Table 2).

**Table 2:** Results of univariable and multivariable Cox regression analysis regarding development of HAI in ICU patients

Variable	Univariable analysis		Multivariable analysis	
	HR (95% CI)	p	aHR (95% CI)	p
Age (per year)	0.98 (0.96–0.99)	**0.02**	0.99 (0.99–1.01)	0.07
Male sex (compared to female)	1.32 (0.71–2.46)	0.40	NA	
SAPS II (per point)	0.99 (0.97–1.01)	0.20	NA	
MV (compared to no MV)	2.09 (0.8–5.41)	0.10	1.73 (0.65–4.61)	0.27
COVID-19 (compared to non-COVID-19)	2.36 (1.38–4.07)	**0.002**	1.87 (1.01–3.47)	**0.045**
COVID-19 density* (per percent)	1.01 (0.99–1.02)	0.10	1.00 (0.99–1.01)	0.75

HAI, health-care associated infections; ICU, Intensive Care Unit; HR, hazard ratio; aHR, adjusted hazard ratio; SAPS II, Simplified Acute Physiology Score II; MV, Mechanical Ventilation; NA, Not Applicable

*defined as weekly percentage of COVID-19 patients among all ICU patients (time-dependent variable)

### Patient mortality

ICU mortality showed no significant difference between COVID-19 positive (13/64, 20.3%) and negative patients (33/190, 17.4%) (*p*-value 0.7). Of the 13 COVID-19 patients who died, 11 (84.6%) had a HAI compared to 8/33 (24.2%) non-COVID-19 patients (chi-square test, *p*-value < 0.01).

## Discussion

In this prospective 12-month HAI surveillance in medical ICU patients, HAIs occurred more often in COVID-19 than in non-COVID-19 patients, even when adjusting for important individual confounders and institutional COVID-19 burden. The observed difference was mainly due to a high incidence of pulmonary infections in COVID-19 patients. These findings indicate that COVID-19 status itself is associated with increased risk for HAI rather than indirect effects of the COVID-19 pandemic such as potential breaches in infection prevention measures.

The 56% of COVID-19 patients experiencing at least one HAI is at the upper limit of what has been described in the literature. In critically ill COVID-19 patients (assessed according to ECDC criteria), numbers range from 26 to 44% [21–23]. Pulmonary infections were the most common HAI in COVID-19 patients. This effect is consistent with existing data [24]. In our study, LRTI rate in COVID-19 patients was 42.8/1,000 patient days. Pneumonia rate was 28.1 per 1,000 ventilator days for COVID-19 patients which is lower compared to a French study (36/1,000 ventilator-days), but in line with data from the UK (28/1,000 ventilator-days) [21, 25]. In comparison to the French study, our COVID-19 patients had a higher SAPS II (43.5 vs. 38) and were more likely to be ventilated (85% vs. 64%), what could have possibly promoted higher infection rates in our patients. BSI was the second most common HAI in our COVID-19 patients, similar to other studies [10, 23]. In fact, our BSI incidence of 9.4% is in line with the 14.9% reported by Buetti et al. [26].

In non-COVID-19 patients, HAI incidence was significantly lower than in COVID-19 patients. This effect was predominantly due to a reduced number of pulmonary infections. BSI incidence was also numerically lower, but did not reach statistical significance, probably due to the small numbers. COVID-19 status remained the only significant factor associated with HAI development in multivariable analysis, which is in line with data from other studies including concurrent non-COVID-19 patients [27]. Whereas the increased HAI risk in COVID-19 patients is intuitive for pulmonary HAI, it is less clear for the similar tendency in BSI. Possible explanations are the high proportion of COVID-19 patients receiving steroid therapy or other anti-inflammatory agents, which predispose for HAI in general. Furthermore, lymphopenia, increased coagulopathy, and bacterial translocation due to mesenteric infarctions associated with severe COVID-19 might play a role, as discussed previously [28].

Of note, HAI incidence in our non-COVID-19 patients was similar to pre-pandemic national prevalence data (20.6%) [29]. Also, we could not demonstrate any influence of the institutional COVID-19 burden on the HAI risk in our patients. These two findings do not support our hypothesis that system-level factors during the COVID-19 pandemic may have contributed to the increased HAI incidence; they rather suggest that the individual COVID-19 status itself is associated with increased HAI risk. These data are in line with a large analysis of over 5 million hospitalizations in the US, which did not find any increase in CLABSI, CAUTI, or bacteremia with methicillin-resistant *Staphylococcus aureus* (MRSA) in non-COVID patients hospitalized during the pandemic [30]. However, the French study by Lepape et al. found VAP and BSI rates among patients without COVID-19 being higher during the pandemic compared to a pre-pandemic control group. They postulate extrinsic factors, such as the breakdown of IPC measures, as possible reasons for elevated HAI risk in non-COVID-19 patients hospitalized during the pandemic [21]. Indeed, understaffing, shortage of personal protective equipment and increased workload during the pandemic have been associated with outbreaks of resistant pathogens in ICUs worldwide [31]. However, our study was performed as of the fourth COVID-19 wave in Switzerland, when fewer patients had to be admitted to the ICU [32]. This could explain why HAI incidence in our non-COVID-19 patients was not elevated compared to pre-pandemic data.

ICU-mortality was similar between COVID-19 (20%) and non-COVID-19 patients (17%). These figures are lower than those reported from the literature, where mortalities of 36% early in the pandemic have been reported in COVID-19 patients on ICUs [33]. Interestingly, almost all COVID-19 patients who died had a documented HAI, which was not the case in non-COVID-19 patients. The causal association between HAI and mortality could however not be assessed with our study design, because of a lack of detailed clinical information such as comorbidities or treatment measures.

The main strength of our study is the availability of prospective surveillance data, which allowed an accurate assessment and encoding of HAI minimizing the risk of reporting bias. Furthermore, we included non-COVID-19 patients as a concurrent control group by which we were able to gain deeper insights into the effects of the pandemic on HAI incidence in both groups. By contrast, being a single-centre experience from a tertiary-care hospital limits the generalizability of our results. Also, the small sample size may have led to reduced statistical power, particularly for the multivariable analysis. Furthermore, our approach of using COVID-19 patient density as a proxy for institutional COVID-19 burden can be debated. Also, further confounding factors, such as understaffing or reduced adherence to hand hygiene, could have biased our results. Finally, since we lacked local pre-pandemic data on specific HAI among ICU patients, we were not able to compare HAI rates in non-COVID-19 patients to local baseline data.

## Conclusion

In conclusion, we observed a higher HAI incidence in COVID-19 than in non-COVID-19 ICU patients, mainly due to an increase in pulmonary infections. Diagnosis of COVID-19 was the only independent risk factor for development of HAI, whereas institutional COVID-19 burden showed no association.

### Electronic supplementary material

Below is the link to the electronic supplementary material.

Supplementary Material 1

## Data Availability

The datasets used and/or analysed during the current study are available from the corresponding author on reasonable request.

## Abbreviations

COVID-19: Coronavirus disease 2019
HAI: Healthcare-associated infections
ICU: Intensive care unit
RR: Risk ratio
CI: Confidence interval
VA-LRTI: Ventilator-associated lower respiratory tract infections
BSI: Blood stream infections
LOS: Length of stay
SARS-CoV-2: Severe acute respiratory coronavirus virus 2
MV: Mechanical ventilation
IPC: Infection prevention and control
SAPS II: Simplified Acute Physiology Score II
DUR: Device utilization ratios
ECDC: European Centre for Disease Prevention and Control
PN: Pneumonia
LRTI: Lower respiratory tract infection
aHR: Adjusted hazard ratios
IQR: Interquartile range
CAUTI: Catheter associated urinary tract infections
CLABSI: Central line associated blood stream infections
MRSA: Methicillin-resistant *Staphylococcus aureus*
