# Supplementary material for: Healthcare‑associated infections in intensive care unit patients with and without COVID-19: a single center prospective surveillance study
Source: Antimicrob Resist Infect Control. 2023 Dec 18;12:147. doi: 10.1186/s13756-023-01353-6 (PMC10729473; doi:10.1186/s13756-023-01353-6)
Supplement: Supplementary file 1 — Supplementary Material 1 [file 13756_2023_1353_MOESM1_ESM.docx]

**Additional file 1**

**Classification of healthcare associated infections**

Bloodstream infection (BSI) refer to primary and secondary laboratory-confirmed BSI. As a central line-associated BSI (CLABSI) is broadly defined as a primary BSI with central line use (even intermittently) in the 48 hours preceding the onset of the infection, we used this term to report central line-related infections (ECDC case definitions C-CVC and CRI3-CVC). For convenience, we reported peripheral catheter/line-related infections with and without microbiologically confirmation (ECDC case definitions C-PVC and CRI3-PVC) both as C-PVC. Secondary BSI are always registered as a separate HAI with exception of CRI3-CVC.

**Figure S1**: Number of healthcare-associated infections (HAI) in COVID-19 vs. non-COVID-19 patients

**Legend figure S1**: VA-LRTI: ventilator-associated lower respiratory tract infections; BSI: blood stream infections (includes CLABSI: central line associated blood stream infections; C-PVC: peripheral vascular catheter infections and secondary blood stream infections); CAUTI: catheter associated urinary tract infections; HAI: healthcare-associated infections.
